# Ultrastructural evidence for completion of the entire miracidial maturation in intrauterine eggs of the digenean *Brandesia turgida* (Brandes, 1888) (Plagiorchiida: Pleurogenidae)

**DOI:** 10.1007/s00436-013-3747-y

**Published:** 2014-02-01

**Authors:** Zdzisław Świderski, Larisa G. Poddubnaya, Aleksander E. Zhokhov, Jordi Miquel, David Bruce Conn

**Affiliations:** 1 W. Stefański Institute of Parasitology, Polish Academy of Sciences, 00-818 Warsaw, Poland; 2 Department of General Biology and Parasitology, Medical University of Warsaw, 5 Chałubińskiego Street, Warsaw, Poland; 3 I.D. Papanin Institute for the Biology of Inland Waters, Russian Academy of Sciences, 152742 Borok, Yaroslavl Province Russia; 4 Laboratori de Parasitologia, Departament de Microbiologia i Parasitologia Sanitàries, Facultat de Farmàcia, Universitat de Barcelona, Av. Joan XXIII, sn, E08028 Barcelona, Spain; 5 Institut de Recerca de la Biodiversitat, Facultat de Biologia, Universitat de Barcelona, Av. Diagonal, 645, E08028 Barcelona, Spain; 6 Department of Biology and One Health Center, Berry College, Mount Berry, GA 30149 USA; 7 Department of Invertebrate Zoology, Museum of Comparative Zoology, Harvard University, 26 Oxford Street, Cambridge, MA 02138 USA

## Abstract

Results of this TEM study provide ultrastructural evidence that miracidial morphogenesis is fully completed within the intrauterine eggs situated in the most posterior uterine regions of the pleurogenid trematode *Brandesia turgida* (Brandes, 1888). The ultrastructural characteristic of different larval organelles and cell types of these eggshell-enclosed, but fully formed, cilated miracidia is described. The body wall of the pyriform mature miracidium of *B. turgida* is composed of ciliated epidermis and underlying peripheral body musculature. Two miracidial flame cells of the protonephridial excretory system are localized in the central region of the ciliated larvae. Three types of miracidial glands were observed: a single apical gland, two lateral glands, and several small vesiculated glands; each gland type contains characteristic, but different types of secretory granules. The anterior end of each miracidium consists of an apical papilla on which are situated the exits of the three main larval glands: an exit of a single apical gland as well as the individual exits of two lateral glands. The exits of vesiculated glands, containing characteristic spherical membrane-bound and highly electron-dense granules, evidently different from the two other types of secretory granules of apical and lateral glands, were not identified. Germinative cells, grouped together in a sac-like germinative follicle, are situated in the medioposterior part of the larva, the germatophore. The germinative cells contain numerous electron-dense heterochromatin islands arranged in the form of a network or chain-like pattern and distributed mainly in the karyoplasm adjacent to the nuclear membrane. The thin layer of granular cytoplasm is rich in free ribosomes and contains a few small mitochondria. Both nuclear and cytoplasmic features if these cells indicate their great developmental potential for further growth and multiplication in postembryonic stages of the life cycle. In the mature eggs, the areas of focal cytoplasmic degradation were frequently observed and may be involved in the autolysis of some embryonic structures. Obtained results are compared with available literature data on the functional ultrastructure of the miracidia of other digeneans.

## Introduction

To our knowledge, there are no published data on the ultrastructure of the intrauterine, eggshell-enclosed mature miracidia of digenean trematodes examined by means of transmission electron microscopy (TEM). Little TEM information is available on the hatched, free-swimming miracidia of different digenean species. More frequent are the scanning electron microscope (SEM) studies on the hatched, free-swimming miracidia of different digenean species (Eklu-Natey et al. 1981, 1985). The TEM studies on digenean eggs have been impeded by serious technical difficulties in getting the egg contents well fixed and infiltrated with embedding media, and also with problems in cutting the thick, hard eggshells. Existing TEM studies have involved mainly parasites of medical or veterinary importance, namely *Fasciola hepatica* (see Wilson 1969a, b, c, 1970, 1971), *Schistosoma mansoni*, *Schistosoma haematobium* (see Świderski et al. 1980; Świderski and Eklu-Natey 1980; Eklu-Natey et al. 1982a, b; Świderski 1984, 1985, 1988, 1994a), *Schistosoma mattheei* (see Świderski 1986), *Schistosoma japonicum* (see Jones et al. 2008) and *Opisthorchis viverrini* (see Khampoosa et al. 2011). The aim of the present study is twofold: (1) to provide ultrastructural evidence for completion of the entire miracidial maturation in the intrauterine eggs of the pleurogenid digenean *Brandesia turgida* and (2) to describe the ultrastructure of these eggshell-enclosed, fully formed, ciliated miracidia of this trematode, a common parasite of the marsh frog *Pelophylax ridibundus* (Pallas, 1771), previously known as *Rana ridibunda*, in Europe. Our previous study on the eggs of this digenean species was focused only on the ultrastructure of the egg wall surrounding the miracidium, with the description of a unique cocoon-like envelope (Świderski et al. 2013a).

## Materials and methods

Adult specimens of *B. turgida* were obtained from the crypts in the intestinal wall of naturally infected frogs, *Pelophylax ridibundus*, collected near the Rybinsk Reservoir on the Volga River, Russia. Live digeneans were rinsed in 0.9 % NaCl solution, fixed in cold (∼4 °C) 2.5 % glutaraldehyde in 0.1 M sodium cacodylate buffer for 10 days, washed overnight in 0.1 M sodium cacodylate buffer at pH 7.4, postfixed in cold (∼4 °C) 1 % OsO_4_ in the same buffer for 1 h, dehydrated in a graded series of ethanol and propylene oxide, and embedded in a mixture of Araldite and Epon. Ultrathin sections were cut on a Leica Ultracut UCT ultramicrotome, collected on copper grids, and double stained with uranyl acetate and lead citrate. Sections were examined in a JEOL 1011 transmission electron microscope operating at 80 kV.

## Results

### General topography of mature miracidia in intrauterine eggs

The miracidia enclosed in the intrauterine eggs of *B. turgida* show the presence of almost all of the larval organelles and cell types characteristic for the mature, ciliated larvae of digeneans, namely: (1) a ciliated tegument; (2) typical miracidial flame cells of their protonephridial system; and (3) different types of miracidial glands such as a single apical gland, two lateral glands and several glandular cells with large, spherical, membrane-bound secretory granules of high electron density, situated in the central region of the miracidium. However, a central nerve ganglion and the sensory nerve endings, characteristic for miracidia of many other digeneans, were never observed in the examined material.

### Ciliated miracidial tegument

**Fig. 1:**
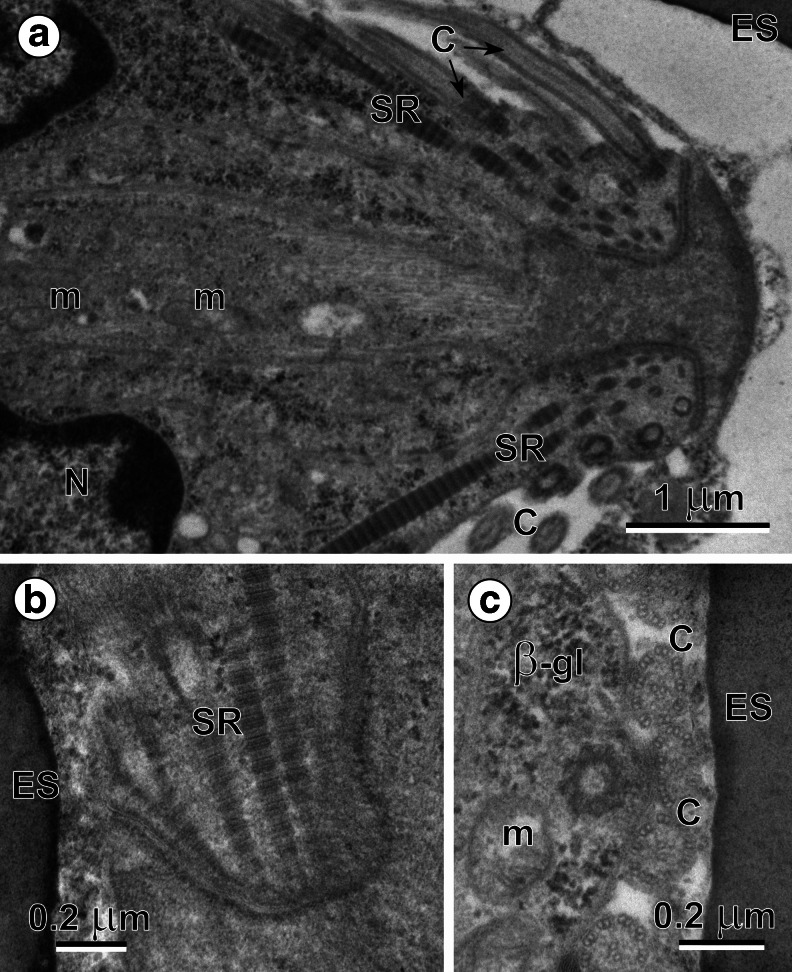
(**a**) The anterior apical region of a mature miracidium of *B. turgida* from the distal part of the uterus. Note the numerous cilia (*C*) and striated ciliary rootlets (*SR*) embedded deeply in the outer cytoplasmic layer of the miracidial tegument. *m* mitochondrion, *N* nucleus. (**b**) High-power micrograph illustrating the ultrastructural details of the striated ciliary rootlets (*SR*). *ES* eggshell. (**c**) High-power micrograph illustrating the ultrastructural details of the 9+2 microtubular pattern of cross-sectioned cilia (*C*) and the nine triplets of the peripheral microtubules on the cross-section made at the level of ciliary basal body in the central part of the micrograph. Note numerous β-glycogen particles (*β-gl*) and several mitochondria (*m*) in the outer cytoplasmic layer of the miracidial tegument. *ES* eggshell

**Fig. 2:**
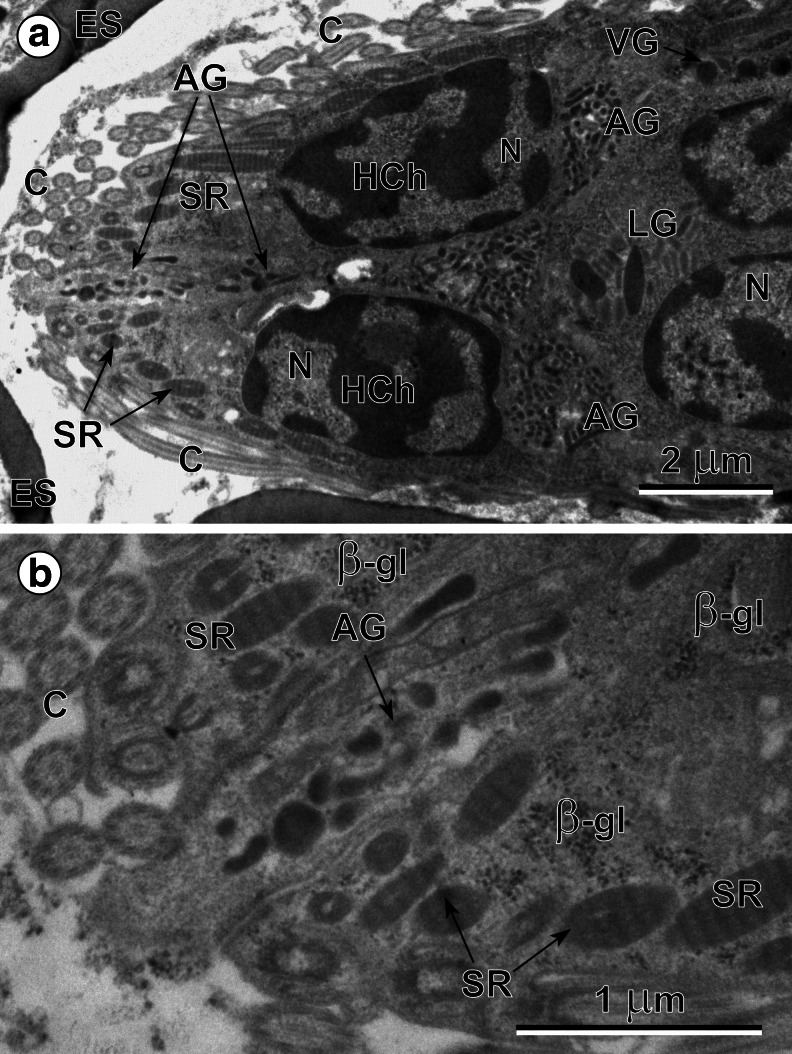
(**a**) The apical region of the miracidium showing the apical gland (*AG*) with its elongated gland exit and a large part of the lateral gland (*LG*) situated behind the apical gland. Note: (1) two large nuclei (*N*) of the apical gland containing much extended, electron-dense heterochromatin islands (*HCh*) of irregular shapes; (2) two large nuclei of the apical gland of similar ultrastructural characteristics: (3) great differences in sizes and shapes of the secretory granules of these two types of miracidial glands: SG-1 of apical gland and SG-2 of the lateral glands. *C* cilia, *ES* eggshell, *SR* striated ciliary rootlets, *VG* vesiculated gland. (**b**) Higher-magnification micrograph showing the details of the apical gland exit (*AG*). Note: (1) numerous moderately electron-densesecretory granules of different shapes and sizes inside of gland exit cytoplasm: and (2) elongated septate junctions between the gland exit and adjacent parts of miracidial tegument containing numerous β-glycogen particles (*β-gl*) and several oblique sections of the striated ciliary rootlets (*SR*). *C* cilia

The body wall of a pyriform mature miracidium of *B. turgida* is composed of a ciliated cellular tegumentary epithelium (Fig. 1), and a very thin layer of underlying peripheral body musculature. The perikarya of the miracidial tegument, situated beneath the peripheral body musculature, are connected with a thin peripheral layer by means of narrow cytoplasmic connections running between the peripheral body musculature. The cilia (Fig. 1a, c), have a typical 9 + 2 arrangement of microtubules with long, striated ciliary rootlets (Figs. 1a and 2b) penetrating deep into the cytoplasm of the epithelial layer. Each cilium starts its growth from the basal bodies (Fig. 1a, c), which below the transition zone shows in cross-section a typical centriolar pattern of nine peripheral triplets in their microtubular arrangement (Fig. 1c).

### Miracidial glands

**Fig. 3:**
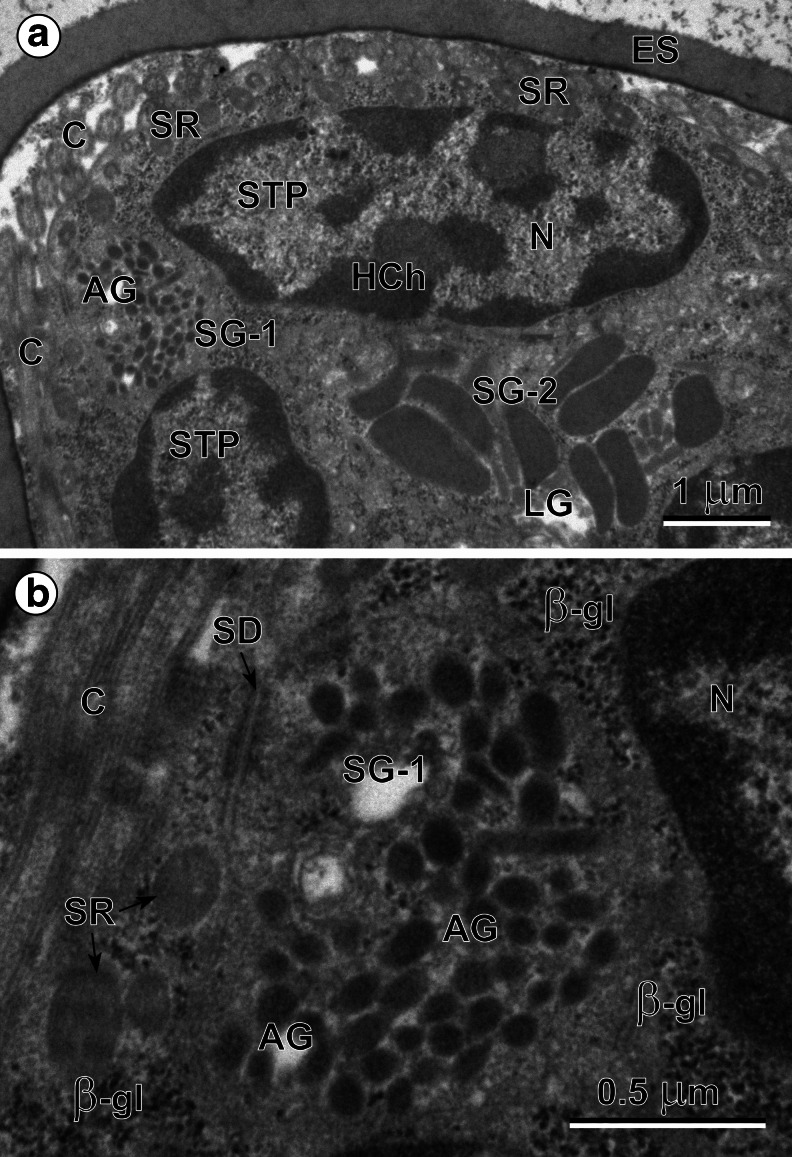
(**a**) Peripheral part of the eggshell-enclosed (*ES*) miracidium showing two large nuclei (*N*) of the tegumental perikarya (*STP*) with numerous heterochromatin islands (*HCh*) of irregular shapes, frequently adjacent to the nuclear envelope. Compare differences in size and shapes of the secretory granules (*SG-1*) of the apical gland (*AG*) with those (*SG-2*) of the lateral gland (*LG*), both situated in small cytoplasmic enclaves of apical and lateral glands between the nuclei of the tegumental perikarya. *C* cilia, *SR* striated ciliary rootlets. (**b**) High-power micrograph of the peripheral region of apical gland (*AG*). Note: (1) numerous β-glycogen particles (*β-gl*) near the gland nucleus (*N*); (2) presence of a septate junction (*SD*) between the apical gland plasma membrane and plasma membrane of the adjacent layer of the ciliated (*C*) miracidial tegument showing several oblique sections of the striated ciliary rootlets (*SR*) and β-glycogen particles. *SG-1* secretory granules of the first type

**Fig. 4:**
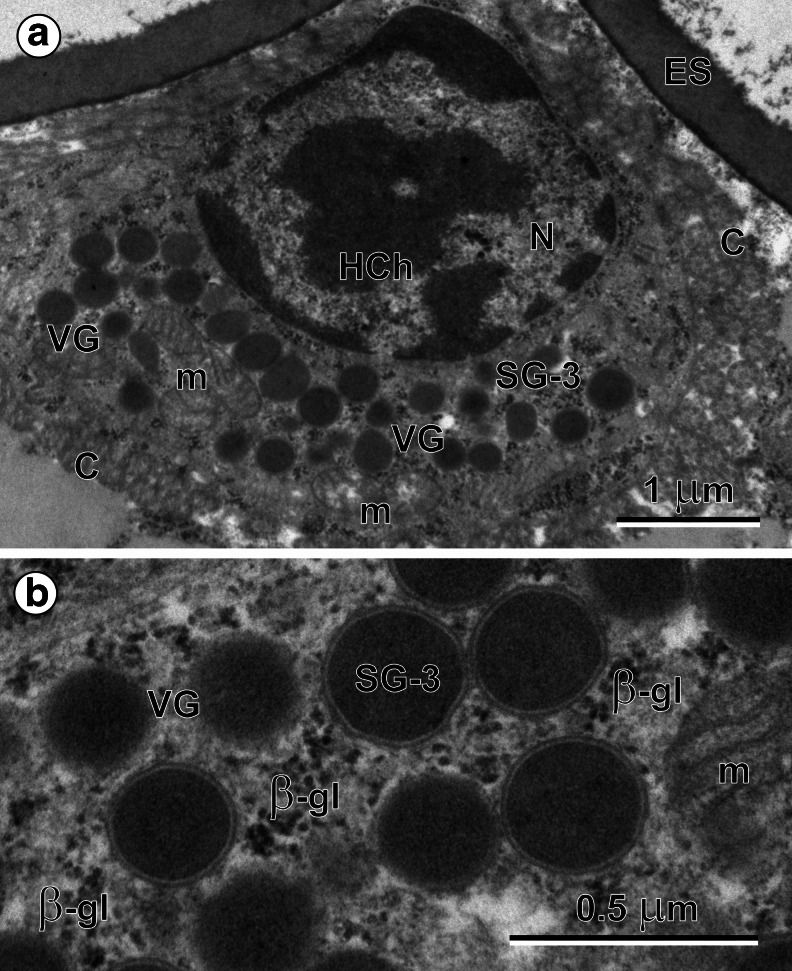
(**a**) Perikaryon of the vesiculated gland (*VG*). Note (1) a large nucleus (*N*) with numerous heterochromatin islands (*HCh*) of irregular shapes in the center of the karyoplasm and around, the nuclear envelope; (2) a high concentration of a spherical, membrane-bound secretory granules of third type (*SG-3*), characteristic for vesiculated gland: and (3) presence of several large mitochondria (*m*) and glycogen particles embedded in the perinuclear granular cytoplasm of the vesiculated gland, rich in free ribosomes. *C* cilia, *ES* eggshell. (**b**) High-power micrograph of the vesiculated gland (*VG*) cytoplasm. Note: (1) the ultrastructural details of a spherical, membrane-bound secretory granules of third type (*SG-3*), (2) large mitochondria (*m*) with elongated, parallel cristae: and (3) randomly dispersed β-glycogen particles (*β-gl*)

**Fig. 5:**
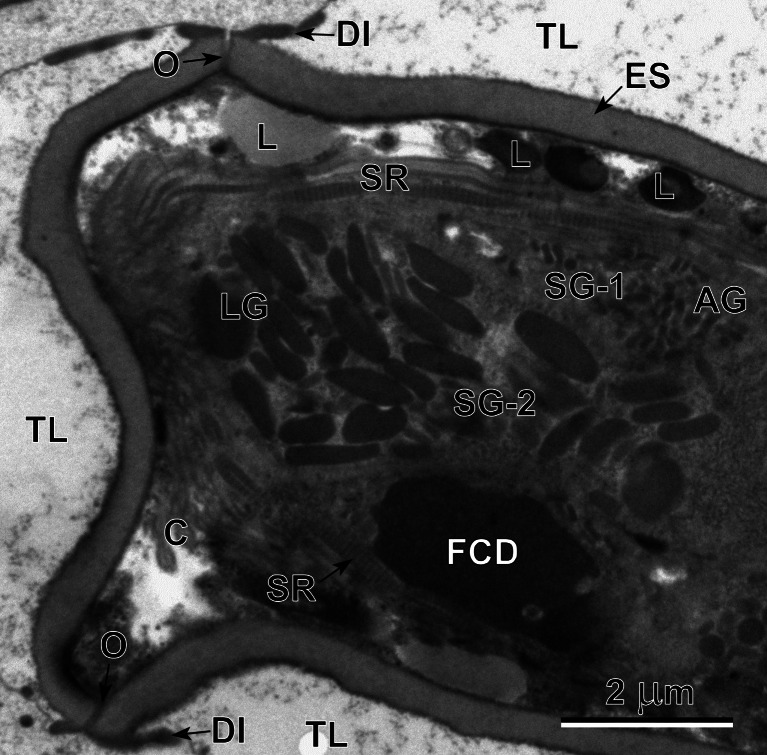
Operculated (*O*) region of the egg showing a part of the lateral gland (*LG*) with a heavy accumulation of the elongated secretory granules of second type (*SG-2*) and adjacent small enclave of the apical gland (*AG*) with its characteristic small secretory granules of the first type (*SG-1*). Compare the sizes and shapes of the secretory granules of these two types of miracidial glands: SG-1 of apical gland and SG-2 of the lateral glands. Note a large electron-dense lysosome-like structure, in the form of areas of focal cytoplasmic degradation in the degenerating miracidial blastomere undergoing apoptosis. *C* cilia, *DI* dense islands of electron-dense material at peripheral membrane of external, electron-lucent cocoon, *ES* eggshell, *FCD* focal cytoplasmic degradation, *L* lipid droplets, *SR* striated ciliary rootlets, *TL* transparent layer of external electron-lucent cocoon

Three types of miracidial glands were observed in these ciliated larvae: a single apical gland (Figs. 2 and 3), two lateral glands (Figs. 2 and 3a) and several small vesiculated glands (Fig. 4); each contains characteristic, but different types, of secretory granules. The anterior end of each miracidium consists of an apical papilla (Fig. 2), on which are situated the exits of the three main larval glands, namely an exit of a single apical gland at the top, as well as the individual exits of two lateral glands, one on each side. The evident differences in size and shapes of two types of secretory granules of the apical and lateral glands are illustrated in Figs. 3a and 5. The secretory granules of the apical gland are much smaller and more electron dense, while those of the lateral are about 20 times bigger and evidently less electron dense. Enlarged details of the perinuclear region of the apical gland granules are illustrated in Fig. 3b. The exits of vesiculated glands were not identified. The large nuclei of these glands show numerous irregularly shaped electron-dense islands of heterochromatin. Their granular cytoplasm, rich in free ribosomes, numerous β-glycogen particles, and large elongated mitochondria, contains a large number of very characteristic spherical membrane-bound and highly electron-dense secretory granules (Fig. 4), which are evidently different from the two other types of secretory granules of the apical and lateral glands.

**Fig. 6:**
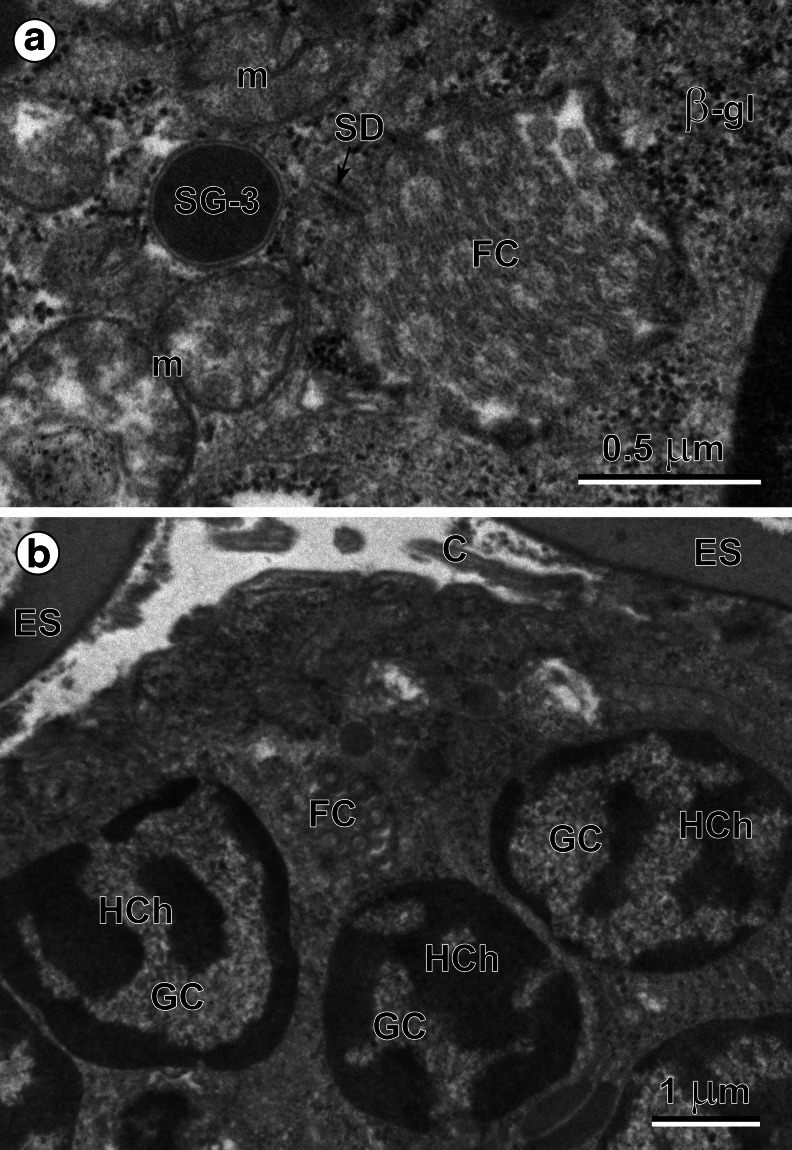
(**a**) Miracidial flame cell (*FC*) cross-sectioned at the level of nephridial funnel. Note: (1) a septate junction (*SD*) in the ring-shaped wall of the nephridial funnel; (2) o cross-section of the flame cell flagella with the typical 9+2 microtubule arrangement; note that the central pairs of microtubules are usually all aligned in the same direction. The vesiculated gland, which surrounds the flame cell show presence of spherical, membrane-bound secretory granules of third type (*SG-3*), characteristic for vesiculated gland, several large mitochondria (*m*) and β-glycogen particles (*β-gl*). Glycogen particles all embedded in the granular cytoplasm of the vesiculated gland, rich in free ribosomes. (**b**) Several germinative cells (*GC*) with large nuclei containing extended electron-dense islands of heterochromatin (*HCh*) of irregular shapes. Note in the central part of the micrograph a cross-section of the miracidial flame cell (*FC*) cut at the level of the nephridial funnel ending, with only a very few flagella of the miracidial flame cell. *C* cilia, *ES* eggshell

**Fig. 7:**
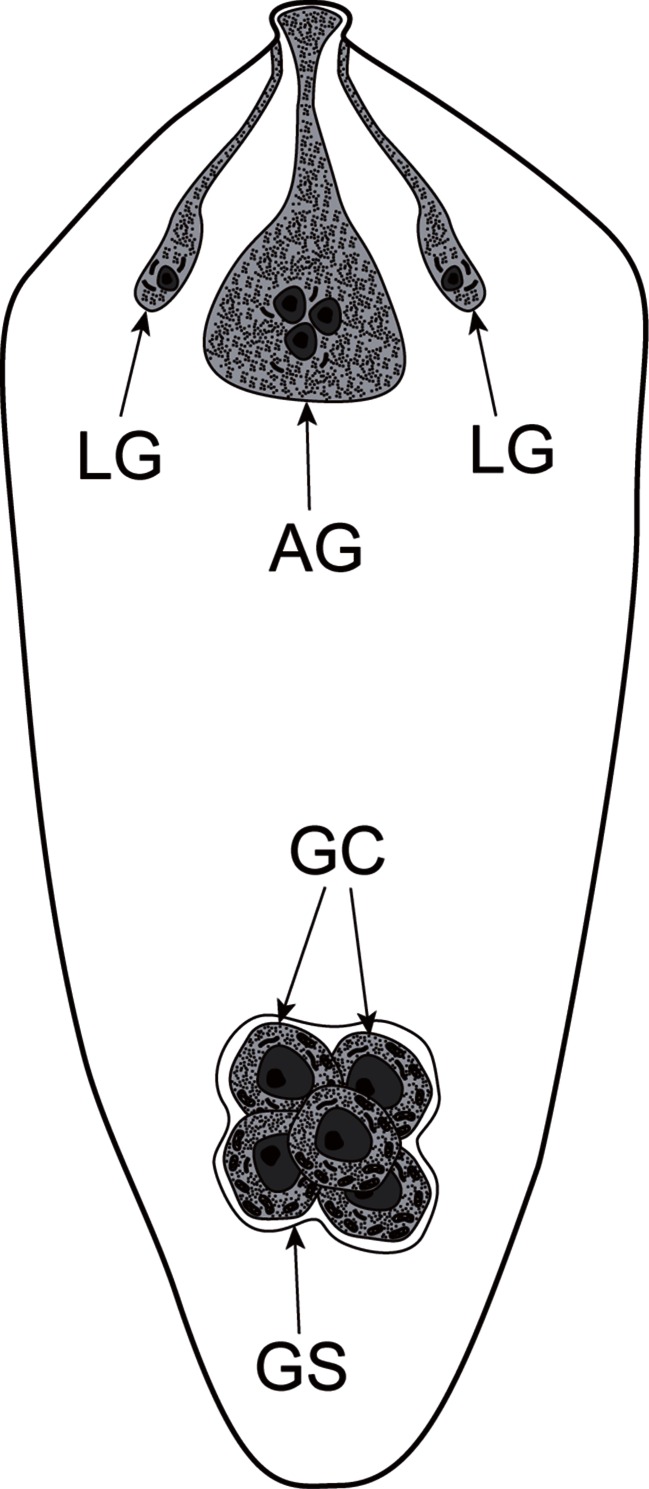
Diagram illustrating position of the germinative cells relative to the glands of the miracidium. *AG* apical gland, *GC* germinative cells, *GS* germinative sac, *LG* lateral glands

### Apoptosis: the degeneration of some blastomeres and a reduction in miracidial cell numbers

The degeneration of some blastomeres, with pycnotic nuclei composed of highly condensed heterochromatin and containing large, electron-dense areas of focal cytoplasmic degradation or lysosome-like structure (Fig. 5) continues in very advanced stages of miracidial maturation, when most of the miracidial structures are already fully differentiated. While several blastomeres, mainly small micromeres, are still undergoing late degeneration, or apoptosis, other organelles of the ciliated larvae already appear functional and are involved in the formation of the different secretory granules characteristic of the three different types of miracidial glands closely adjacent to the degenerating blastomere containing a large, electron-dense lysosome-like structure (Fig. 5).

### Flame cells of the protonephridial system

The protonephridial system of the miracidium is composed of two terminal organs (i.e., flame cells) with two small collecting ducts that run from each flame cell towards the excretory pore (i.e., nephridiopore). The cross-section of the nephridial cell cut at the level of the nephridial funnel (Fig. 6a), forms a ring-like structure, the concave surfaces of which are connected by a circular, septate junction (Fig. 6a). The flame cell flagellar tuft, which lies within the lumen of the nephridial chamber and nephridial funnel, is composed of about 25 flagella. Their number, however, when observed on different cross-sections, varies greatly among cross-section levels. In cross-section, the flame cell flagella have the typical 9 + 2 microtubule arrangement (Fig. 6a). The central pairs of microtubules are usually all aligned in the same direction (Fig. 6a), apparently transversely to the beat of the flagella. All flagella are tightly packed together (Fig. 6a) indicating that the flagellar tuft (i.e., “flame”) might function as a single entity.

### Germinative cells

The germinative cells are grouped together in a sac-like germinative follicle, situated in the medioposterior part of the larva, the germatophore. It consists of numerous germinative cells and a few irregularly shaped interstitial cells. The miracidial germinative cells of *B. turgida* are ovoid or slightly angular in shape (Fig. 6b). Each has a large, spherical nucleus, about 7 μm in diameter, which occupies more than half of the cell volume (Fig. 6b). They contain numerous electron-dense heterochromatin islands arranged in the form of a network with a chain-like pattern and distributed mainly in the karyoplasm adjacent to the nuclear membrane (Fig. 6b). The thin layer of granular cytoplasm is rich in free ribosomes and contains a few small mitochondria (Figs. 6b and 7).

### Apoptosis in embryonating and embryonated eggs

Both embryonating and embryonated eggs of *B. turgida* show evident signs of degeneration of some blastomeres and/or undifferenciated miracidial cells still undergoing late apoptosis. Such disintegrating cells show the presence of a large electron-dense lysosome-like structure, in the form of areas of focal cytoplasmic degradation (Fig. 5), which are always characteristic features of such degenerating miracidial cells/blastomeres undergoing apoptosis.

## Discussion

### Oviparity, ovoviviparity, viviparity, and hatching in parasitic Platyhelminthes

It is possible that the eggs of *B. turgida*, with a quite unusual type of operculum, must be ingested by a specific first intermediate host. The intrauterine eggs of *B. turgida* greatly resemble those of *Mediogonimus jourdanei* (Świderski et al. 2010) as in both of these digenean species contain already mature, ciliated miracidia, apparently ready for hatching, which seems to take place immediately after egg evacuation into the external aquatic environment. It looks therefore that such a very advanced degree of ovoviparity as observed in these two digeneans is very close to viviparity, with their intrauterine eggs containing fully formed ciliated miracidia; however, it cannot yet be classified as viviparity, because the larval hatching still takes place in water. In digeneans, hatching of eggs in some very rare cases occurs within the uterus of the adult worm, e.g., *Parorchis acanthus* and *Philophthalmus hegeneri* (see Fried and Haseeb 1991); this can be classified according to some authors as examples of viviparity (Fried and Haseeb 1991, p. 160). Typical cases of viviparity take place, however, rather seldom. Examples were reported in a comparative study describing the ultrastructural adaptations for viviparity in the female genital system of three species of gyrodactylid monogeneans (Cable et al. 1996), demonstrating in each of them a similar pattern of intrauterine development. These viviparous gyrodactylids have a unique mode of reproduction, in which several generations of embryos develop inside each other, with each successive generation nested inside its parental generation, and with all embryo nutrients being passed across the uterine wall from the mother. However, other species of monogeneans also have intrauterine development of larvae (Tinsley 1983; Cable and Tinsley 1991; Cable et al. 1997), with apparent uterine contribution to embryonic or larval nutrition. Future studies should examine comparisons between these and the aspidogastreans, digeneans, and cestodes that exhibit strategies ranging from oviparity to ovoviviparity, as well as the possibility of viviparity in other parasitic flatworm taxa. In particular, the variations in maternal characteristics such as uterine structure and development may reflect differences in the maternal strategies in establishing larval readiness for progression to the next stage of the life cycle (Conn 1987, 1993; Conn and Forman 1993; Conn et al. 2009; Świderski et al. 2012). The early eggs of *B. turgida*, like those of *Maritrema feliui* (Świderski et al. 2011a, 2013b), in comparison with the early eggs of two species of bothriocephalidean cestodes *Bothriocephalus clavibothrium* (Świderski and Mokhtar 1974; Świderski 1994b; Świderski and Mackiewicz 2007a, b) and *Clestobothrium crassiceps* (Świderski et al. 2013c) as well as caryophyllidean cestode *Khawia sinensis* (Bruňanská et al. 2012) contain a much smaller number of vitellocytes per fertilized ovum. However, during the in utero development of *B. turgida* eggs, both nutritive and protective functions of the vitelline cells and embryonic envelopes are taken over by the uterus. This may explain why these two functions of vitellocytes may be much intensified or reduced to some or to great extent in trematodes and cestodes with entirely intrauterine, partially intrauterine or entirely free embryonic development taking place in the external aquatic environment during their life cycles.

### Ciliated miracidial tegument

The ciliated tegument of the larvae of *B. turgida* generally resembles that of other digenean miracidia (Wilson 1969a, b; Pan 1980; Eklu-Natey et al. 1981; Świderski 1984; Eklu-Natey 1986) and of lower cestodes: lycophores (Xylander 1986, 1987, 1990, 2001); or coracidia (Świderski 1994a, c; Świderski and Mackiewicz 2004). However, the miracidial tegument of *B. turgida*, when examined in eggshell-enclosed miracidia, in contrast to those of hatched miracidia of schistosomes, shows no subdivision into ciliated epidermal plates, arranged in four tiers and consisting of different number of plates in different species, and separated longitudinally by intercellular, non-ciliated ridges. In *B. turgida* eggshell-enclosed miracidia, it is simply composed of an anucleate layer of peripheral cytoplasm covered by a great number of cilia with long, periodically striated ciliary rootlets deeply embedded in the tegumental cytoplasm. It is separated from the very thin layers of the peripheral musculature by a basal matrix. The tegumental perikarya are situated beneath the peripheral musculature and connected to the peripheral ciliated layer of tegument by short cytoplasmic connections or bridges as described previously in the miracidia of schistosomes (Pan 1980; Eklu-Natey 1986; Świderski 1984, 1985) or liver flukes (Wilson 1969a, b).

### Protonephridial system

The protonephridial system of *B. turgida* miracidia resembles, to a great extent, that described in ciliated larvae of lower cestodes, such as lycophores (Xylander 1986, 1987, 1990, 2001) and coracidia (Świderski 1994b; Świderski and Mackiewicz 2004), or in adult cestodes (Świderski et al. 1973). Circularly arranged, minute pores or nephrostomes, as described from three species of cyclophyllideans (Świderski et al. 1973), occur between the endings of the outer row of digitiform processes arising from the nephridial funnel and the surface of the leading edge of the flame cell. These pores provide a direct communication between the nephridial chamber and intercellular regions of the medullary parenchyma and, therefore, play an important role in the filtration apparatus.

### Germinative cells

The germinative cells of digenean miracidia are easily recognized by their large nuclei which occupy nearly one half of each cyton, and by the numerous electron-dense heterochromatin islands accompanied by their nucleoli of different sizes and electron density in different species. In *B. turgida*, as in *S. mansoni* (Pan 1980; Świderski 1984, 1985) and *S. haematobium* (Świderski and Eklu-Natey 1980; Eklu-Natey 1986) these are localized in the medioposterior parts of their ciliated larvae. The lobate nuclei of their cytons in the miracidia of *B. turgida* are of similar size or slightly larger than in two above mentioned schistosome species. In *S. mansoni*, according to Pan (1980), they may measure 5 μm and in *S. haematobium* about 7 μm (Świderski and Eklu-Natey 1980) at their widest diameter. The large number of free ribosomes in their cytoplasm, described both in digenean miracidia and in cestode coracidia and oncospheres (Świderski et al. 2002), is an indication of their capability to synthesize large amounts of proteins for use in situ. Olivier and Mao (1949) described an average of 53 germinative cells in a miracidium, and 200–400 germ balls in the 14-day-old mother sporocysts. Their observations suggest that the germinal cells multiply rapidly in the mother sporocyst after the miracidium enters a suitable snail host, and that the numerous ribosomes in the cytoplasm function actively in protein synthesis, necessary for cell division.

Both nuclear and cytoplasmic features observed in the ultrastructure of germinative cells within the miracidium may indicate their great developmental potential for further growth and multiplication. It is evident that they play an important role in digenean ontogenesis, as by definition they are involved in a growth and differentiation of the sporocyst, the following stage of the parasite life cycle.

### Apoptosis in embryonating and embryonated eggs

In *B. turgida*, apoptosis was also frequently observed and described in our previous paper (Świderski et al. 2013a) both in the early embryos and in degenerating egg envelopes. Apoptosis at early stages of development was visible in all blastomeres and resulted in entire egg degeneration or autolysis. Regarding egg envelopes, the lysosome-like structures, i.e., area of focal cytoplasmic degradation, were localized in the cytoplasm of the inner envelope of the maturing and mature eggs. As initially reported after a light microscope study by Rybicka (1961) and confirmed at the TEM level by Świderski (1968), the apoptosis or degeneration of numerous micromeres culminates in a reduction in the number of cells in the resulting larva. This is a common feature for both lower (Świderski 1994b; Świderski and Mackiewicz 2004; Młocicki et al. 2010) and higher cestodes (Świderski 1968, 1981; Conn and Świderski 2008), and also occurs in aspidogastreans (Świderski et al. 2011b, 2012) and schistosome digeneans that have been investigated (Świderski 1984, 1985, 1994a, c).

### Suggestions on functional ultrastructure of miracidia and working hypothesis on *B. turgida* life cycle

The life cycle of *B. turgida* is unknown. The eggs of some digeneans hatch only if/when ingested by specific snail first intermediate hosts, e.g., *Haematoloechus medioplexus* and *Clonorchis sinensis* (Fried and Haseeb 1991). Also, mature eggs of *M. feliui* can hatch only when ingested by specific snail hosts (Villa 2007). Egg ingestion was also described in the case of *Echinostoma caproni* by Idris and Fried (1996), during experimental infestation of *Biomphalaria glabrata* snails fed with embryonated eggs of *E. caproni.* It is possible that cases of hatching that occurs only after egg ingestion by a specific snail host may also occur in the pleurogenid *B. turgida.* Unfortunately at this stage, such a mode of infection by ingestion of the entire egg of *B. turgida* could only be considered as one of our working hypotheses for interpretation of the functional ultrastructure of eggs and eggshell-enclosed, mature miracidia of *B. turgida*. Such a hypothesis could eventually explain why in the miracidia of *B. turgida*, we have never seen any traces of the nerve ganglion or any type of the sensory nerve endings. If they are hatching only when ingested by a specific snail, this would represent a passive way of host infection comparable to passive penetration of coracidia of *B. clavibothrium* after ingestion by their copepod hosts (Świderski 1994b). In such a case, it could be easily explained why they do not need sensory nerve endings or nerve ganglia. Such structures may be useful only during an active mode of host invasion, for active detection of their specific snail host. Another possible hypothesis for explaining lack of any nerve structures in the intrauterine, eggshell-enclosed miracidia of *B. turgida*, which cannot be excluded, is a rapid formation of nerve ganglion and sensory nerve endings just before or during hatching of miracidia. However, this second hypothesis, in our opinion, seems to be even less probable. Further study of this species along with others with similar life modes of invasion are recommended to test this hypothesis. In addition, the presence of a unique cocoon-like structure on the surface of the eggshell, which apparently increases its volume in water (Świderski et al. 2013a), could eventually play an adaptive role of egg adaptation resulting in a very attractive mimicry that increases the chances of ingestion of *B. turgida* eggs by its snail host.
